# Effects of aging on host responses in gingival crevicular fluid in natural gingivitis

**DOI:** 10.3389/fimmu.2026.1761707

**Published:** 2026-02-26

**Authors:** Yung-Ting Hsu, Jonathan Y. An, Johnny Hudson, Diane Daubert, Richard P. Darveau

**Affiliations:** 1 Department of Periodontics, University of Washington School of Dentistry, Seattle, WA, United States; 2 Department of Oral Health Science, University of Washington School of Dentistry, Seattle, WA, United States

**Keywords:** age, cytokines, gingivitis, immune system, metalloproteinases

## Abstract

**Aim:**

This study compared host responses against natural gingivitis in the gingival crevicular fluid (GCF) in young and aged periodontium.

**Materials and methods:**

This cross-sectional study recruited two patient populations with natural gingivitis in young (18–35 years old) and elder cohorts (36–75 years old). GCF samples were analyzed for 39 inflammatory and tissue remodeling mediators using bead-based multiplex immunoassays. Independent t-tests with false discovery rate adjustments were used to compare mediator expressions between groups.

**Results:**

Forty patients were enrolled in these comparisons, including young patients (n=22) and elder patients (n=18). In comparison with the young group, the elder group had greater clinical attachment loss and higher expression of MPO (p<0.001), IL-1β (p<0.05), IL-6 (p<0.05), and IL-8 (p<0.05). Despite the ratio of MMP-8/TIMP-1 being not significantly different (p>0.05), the young group had greater ratio of MMP-9/TIMP-1 than old group (p<0.01).

**Conclusion:**

The current findings reveal that the inflammatory profiles of young and elder cohorts with natural gingivitis are distinct. The young cohort with natural gingivitis demonstrated lower disease susceptibility and more robust tissue turnover, whereas the aged periodontium was characterized by higher susceptibility to disease and diminished healing capacity.

## 1 Introduction

Aging is characterized by a progressive and persistent decline in intrinsic physiological functions (1). Within the periodontium, aging affects homeostatic regulation mechanisms and alters molecular profiles, influencing how periodontal tissues respond to microbial challenges (2). Indeed, age-related changes distinctly affect both innate and adaptive immune responses, leading to immunosenescence and inflammaging, i.e., the phenomena associated with increased susceptibility to infection and heightened proinflammatory status (3).

Evidence from human experimental gingivitis models reveals that age significantly modulates host defenses during the induction of gingival inflammation (4). Studies comparing young (20–25 years old) and older adults (average age more than 55 years old) showed that the young population had less plaque accumulation and gingival inflammation clinically (5–7). Compared with the young group, older adults showed greater expression of pro-inflammatory mediators and tissue remodeling (6–8).

Although the severity and extent of periodontitis are widely recognized to increase with age, few studies have explored the impact of aging on natural gingivitis, the early stage of periodontal inflammation. Induced by bacterial dental plaque, natural gingivitis leads to a cascade of events affecting immune and tissue homeostasis in periodontium, which is associated with further tissue breakdown and disease progression (9). Understanding age-related differences in gingival inflammation will provide insights into susceptibility to periodontitis and facilitate tailoring individualized treatment strategies. Therefore, this study aims to explore the differences in gingival inflammation between young and elder populations by assessing their clinical outcomes and host defenses, in terms of immune regulation and tissue remodeling, in gingival crevicular fluid (GCF).

## 2 Materials and methods

The study was approved by the human subject’s ethics board of the University of Washington (UW IRB# STUDY00012410) and was conducted in accordance with the Helsinki Declaration of 1975, as revised in 2013. This human observation study complied with the STROBE guideline.

### 2.1 Patient inclusion and exclusion criteria

This cross-sectional study recruited two patient populations with natural gingivitis in young (Y) group, defined as age of 18–35 years old, whereas the Elder (E) group enrolled patients with 36–75 years old. Participants were categorized into two age groups based on both clinical and cohort-specific considerations. The cutoff of 35 years was selected in alignment with the clinical definition of early-onset periodontitis, which is typically diagnosed before age 35 (10, 11. Additionally, the age distribution within our cohort demonstrated a natural separation between individuals ≤34 years and ≥38 years, with no participants in the intermediary range. The age gap arose naturally because our school–based clinic primarily attracts university students and older adults with more flexible schedules. This data-driven approach minimized misclassification and avoided imposing arbitrary thresholds. Using this criterion allowed us to capture individuals at a biologically relevant transition point where age-related changes in immune function and periodontal tissue may begin to emerge.

Inclusion criteria: Patients should have generalized gingivitis following the definition of the 2018 AAP/EFP disease classification without any history of periodontitis (12).

Exclusion criteria included: (1) use of antibiotics or nonsteroidal anti-inflammatory medications within 30 days, (2) patients with ASA I and II (13), (3) refusal to sign the informed consent or unable to communicate in English, (4) patients who received active periodontal therapy such as scaling and root planning, and periodontal surgery within the last 2 years.

### 2.2 Calibration

All patient recruitment, examination and GCF sample collection were done by a single examiner (YH). The intra-examiner calibration process was performed on five volunteers not enrolling in this study prior to the beginning of the study. The same quadrant was probed two times separately between 1–3 weeks apart. The intra-examiner agreement was achieved to ≥ 80% before the beginning of the study.

### 2.3 Clinical examination and GCF collections

Patients were screened after informed consent was signed. Demographic information and history of medical and medication history were reviewed and recorded, including age, gender, smoking habit, and health status. Periodontal examination was further performed, recording the following clinical indices, including clinical attachment level (CAL), probing depth (PD), bleeding on probing (BOP), visible plaque index (PI) (14), and gingival index (GI) (15). GCF samples and clinical indices were collected at 6 surfaces of 6 selected teeth. All GCF samples were collected using sterile paper strips for 30 seconds (Periopaper; Oraflow Inc, Smithtown, NY, USA) into the gingival crevice and stored at -80°C until processing (16).

### 2.4 GCF samples analysis

All GCF samples underwent an assay of 39 mediators of inflammatory/immune responses and tissue remodeling using commercially available bead-based multiplex immunoassays (Procartaplex 39-plex Panel, Thermo Fisher Scientific, Waltham, MA, USA). The GCF samples were extracted using 200 µl of 0.01M PBS to each tube and rotating for one hour at 4°C. An aliquot of 10µl per sample was transferred for protein analysis (VMax microplate reader; GMI, Ramsey, MN, USA). Based on the protein concentration, the GCF samples were diluted with 0.01M PBS to normalize samples to 10µg/ml. Bead based multiplex analysis was then performed on duplicated, standardized GCF samples (i.e., samples diluted to 10µg/ml) using the bead-based multiplex immunoassays according to manufacturer’s protocol. The data was obtained using a flow-cytometry-based array reader (Bio-Plex 200 reader, Bio-Rad Laboratories, Hercules, CA, USA), by acquiring the signal from the fluorescent dye within each bead for assay identification along with the fluorescent signal from the reporter for quantification. The data was analyzed with a software (Bio-Plex Manager Software V6, Bio-Rad Laboratories, Hercules, CA, USA) and the concentrations of different mediators were calculated based on the respective standard curve for each chemokine with 5-parameter logistic (5PL) equation. Mediator data are reported in mean value with the unit of pg/ml. The intra-plate variability is 7.75% in average (6.33-9.64%) and the inter-plate variability is 5.02%.

To better characterize the disease processes relevant to aging and periodontal inflammation, we selected a panel of biomarkers representing key biological pathways implicated in mucosal immune regulation and tissue turnover. Mediators associated with angiogenesis (angiogenin, angiopoietin–1, angiostatin, vascular endothelial growth factor/VEGF–A) were included because vascular adaptation is essential for sustaining inflammatory cell infiltration and tissue remodeling. Biomarkers involved in innate and adaptive immune responses (C3a, C–reactive protein/CRP, ENA–78/CXCL5, fractalkine/CX3CL1, GCP–2/CXCL6, granulocyte–macrophage colony–stimulating factor/GM–CSF, intercellular adhesion molecule–1/ICAM–1, interleukin/IL–1β, IL–6, IL–8, IL–12p70, IL–17A, LOX–1, MCP–1/CCL2, macrophage migration inhibitory factor/MIF, MIP–1α/CCL3, CCL5/RANTES, stromal–derived factor–1α/SDF–1α, and myeloperoxidase/MPO) were selected to evaluate neutrophil activity, chemotactic responses, and cytokine-driven inflammation. Markers of tissue remodeling (metalloproteinase/MMP–1, MMP–2, MMP–3, MMP–7, MMP–8, MMP–9, MMP–12, MMP–13, osteopontin/OPN, osteoprotegerin/OPG, receptor activator of NF–κB ligand/RANKL, and tissue metallopeptidase inhibitor 1/TIMP–1) were included to detect dysregulated extracellular matrix turnover and bone resorption as the hallmark events in periodontal aging and disease. Finally, C–peptide and insulin were assessed for their potential relevance to systemic metabolic status, which may influence inflammatory responses in the periodontium.

### 2.5 Statistical analysis

The data of all mediators and clinical parameters were analyzed using a series of independent samples t-tests using GraphPad Prism (Vers. 10.6.1) and R (17, 18). Multiple comparisons were corrected using the Benjamini-Hochberg formula for the False Discovery Rate (FDR) correction (19). Several analytes were flagged as out of range (“<OOR”), indicating concentrations below the assays’ lower limit of quantification (**Supplementary Table S1**). These values were set to zero to reflect that protein levels were too low for reliable detection using this assay. As an exploratory pilot study, a formal power calculation was not conducted prior to recruitment.

## 3 Results

### 3.1 Demographic and clinical information

A total of 40 patients were recruited in this study, including 22 patients in Y group (15 females and 7 males) and 18 patients (13 females and 5 males) in E group. The average ages of Y and E groups were 26.82± 3.76 and 60.5± 13.35 years old. Only 1 patient in E group reported the history of controlled diabetes (HbA1C level=7%) and the rest of the patients reported no history of diabetes. According to the patients’ self-reports, Y group consisted of 15 never smokers, 2 former smokers and 5 current smokers. E group had 13 never smokers and 5 former smokers.

**Figure 1:**
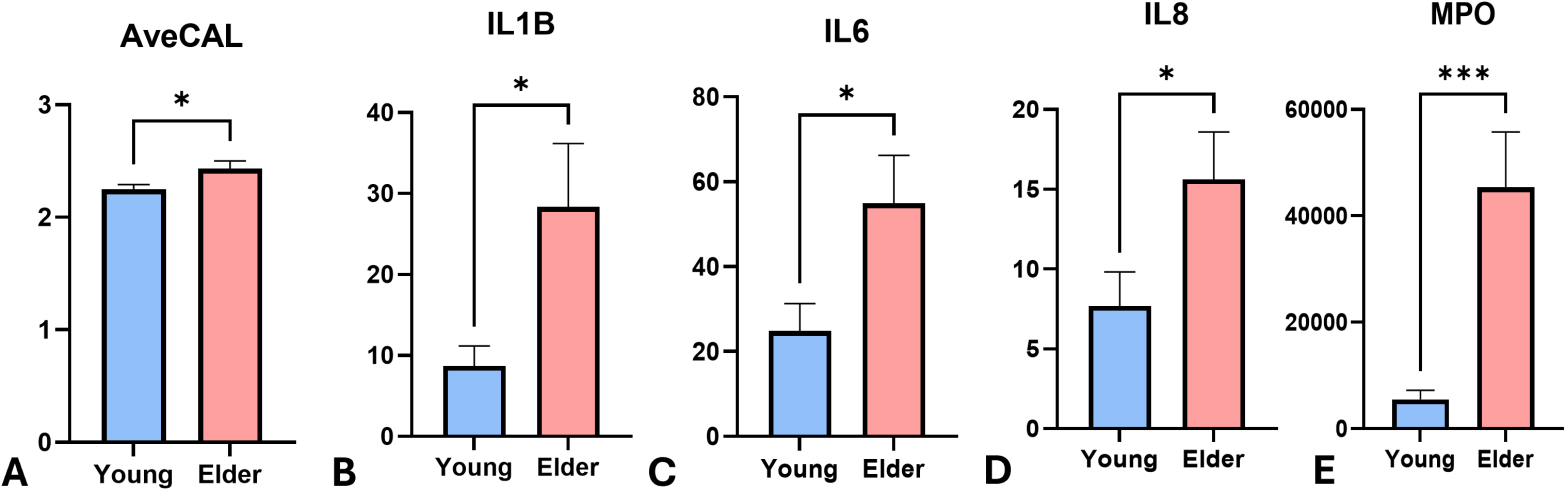
Levels of clinical attachment and immune regulatory mediators between 2 groups. The expression level of **(A)** average clinical attachment level (AveCAL) **(B)** IL-1β **(C)** IL-6 **(D)** IL-8 **(E)** MPO comparing young (Y) (n=22) and elder **(E)** cohorts (n=18). Boxes represent data and medians ± interquartile ranges (IQR); whiskers and outliers > 1.5 IQR below (above) the 25th (75th) percentile. The means were presented in the gray triangles. Differences among each group are shown above the groups and their significance level indicated by asterisks. Significance levels: *P < 0.05, and ***P ≤ 0.001.

All clinical and demographic information were summarized in **Table 1**. Average CAL differed significantly between groups (p<0.05, **Figure 1A**), with the Y group at 2.25± 0.19mm and the E group at 2.43± 0.20mm. There was no significant difference in the rest of the clinical parameters, including PD, GI, and PI (p>0.05).

**Table 1:** Comparison of demographics and clinical parameters between groups.

Variables	Young group (n=22)	Elder group (n=18)	p-value
Demographics/mean (SD)			
Age (y/o)	26.82 (3.76)	60.5 (13.35)	
Gender	15 females and 7 males	13 females and 5 males	
Clinical Parameters/mean (SD)			
Probing depth (mm)	2.27 (0.21)	2.00 (0.20)	p>0.05
Clinical Attachment Level (mm)	2.25 (0.19)	2.43 (0.20)	p<0.05
Plaque index	1.20 (0.34)	1.12 (0.34)	p>0.05
Gingival index	0.60 (0.45)	0.74 (0.46)	p>0.05

### 3.2 The expression of mediators associated with immune regulation

**Table 2** and **Figure 1** summarized the comparison of immune regulatory mediators between the 2 groups. In comparison of Y group, E group had greater levels of LOX-1 (p<0.05), an inflammation amplifier, and greater expressions of pro-inflammatory mediators, such as IL-1β (p<0.05, **Figure 1B**), IL-6 (p<0.05, **Figure 1C**), IL-8 (p<0.05, **Figure 1D**), IL-17 (P<0.01), MIF(p<0.05), and CCL3 (p<0.01). Y group had lower levels of neutrophil related mediators, such as MPO (p<0.05, **Figure 1E**), ICAM-1 (p<0.05), and CXCL5 (p<0.05) when compared to the E group. The expression of fractalkine (p<0.05), GM-CSF (p<0.05), and CCL2 (p>0.05), which were associated with the activities of monocyte and macrophage, were lower in Y group compared to E group. C3a, CRP, ENA-78/CXCL5, and CXCL6 were also tested but did not show significant differences between groups (p>0.05, **Table 2**).

**Table 2:** Comparison of mediator expressions in original scales.

Mediators	Young (Y) (n=22)	Elder (E) (n=18)	P-Value	FDR Adjusted P-Value
LOX_1	27.9	57.5	0.035	0.059
IL-1B	8.7	28.3	0.013	0.049
IL_6	24.9	54.9	0.019	0.049
IL_8	7.6	15.6	0.032	0.059
IL_17A	11.6	28.1	0.002	0.021
MIF	26.8	73.2	0.018	0.049
CCL3	0.3	4.9	0.007	0.033
MPO	5530.3	45373.5	0.001	0.003
ICAM_1	539.1	1339.9	0.021	0.049
CCL5/RANTES	2.3	5.5	0.030	0.059
Fractalkine/CX3CL1	0.8	1.8	0.034	0.059
GM_CSF	27.9	60.7	0.031	0.059
CCL2	4.9	15.9	0.004	0.027
C3a	310.4	397.6	0.557	0.583
CRP	54.2	67.6	0.654	0.661
ENA-78/CXCL5	9.7	43.7	0.022	0.049
CXCL6	0.3	0.6	0.152	0.201
Angiostatin	31.2	82.2	0.021	0.049
IL_12p70	6.5	19.5	0.004	0.026
SDF_1_alpha	31.9	94.6	0.019	0.049
Angiogenin	0.8	1.2	0.271	0.330
Angiopoietin-1	24.3	34.8	0.453	0.497
VEGF_A	20.4	39.9	0.055	0.083
MMP_1	58.3	78.5	0.264	0.330
MMP_2	21.1	98.5	0.007	0.033
MMP_3	9.6	33.3	0.008	0.033
MMP_7	131.6	49.3	0.044	0.070
MMP_8	825.1	1516.7	0.113	0.156
MMP_9	11254.9	1721.9	0.001	0.020
MMP_12	80.5	96.5	0.661	0.661
MMP_13	363.0	206.1	0.303	0.359
TIMP_1	260.5	705.2	0.002	0.021
OPN	71.5	254.2	0.001	0.001
OPG	0.1	2.0	0.054	0.083
RANKL	4.4	12.1	0.033	0.059
BMP-2	567.1	1122.0	0.159	0.205
Insulin	954.2	1914.5	0.028	0.059
c-peptide	71.5	134.2	0.003	0.026

Protein concentration of standardized mediators (pg/ml) was described by mean. The P-value and FDR adjusted P-Value were also included for the comparisons between groups.

CR, C-reactive protein; GM-CSF, Granulocyte-macrophage colony-stimulating factor; ICAM-1, intercellular adhesion molecule-1; IL, Interleukin; MMP, matrix metalloproteinase; MPO, myeloperoxidase; OPN, osteopontin; OPG, osteoprotegerin; RANKL, Receptor activator of nuclear factor-κB ligand; TIMP, metallopeptidase inhibitor; VEGF, Vascular endothelial growth factor.

### 3.3 The expression of mediators associated with angiogenesis and tissue turnover

**Figure 2:**
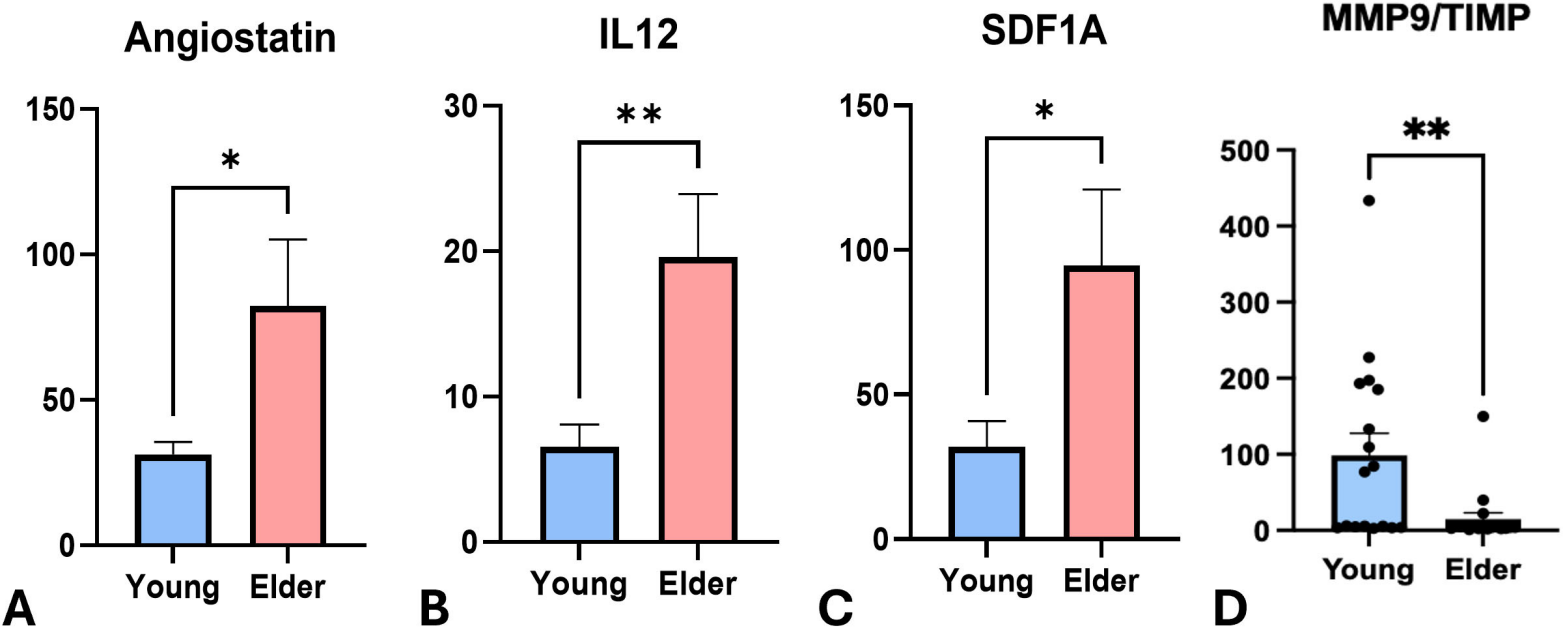
Levels of mediators associated with angiogenesis and tissue remodeling between 2 groups. The expression level of **(A)** Angiostatin **(B)** IL-12 **(C)** SDF-1A **(D)** MMP-9/TIMP-1 comparing young (n=22) and elder cohorts (n=18). Boxes represent data and medians ± interquartile ranges (IQR);whiskers and outliers > 1.5 IQR below (above) the 25th (75th) percentile. The means were presented in the gray triangles. Differences among eachgroup are shown above the groups and their significance level indicated by asterisks. Significance levels: *P < 0.05, and **P ≤ 0.01.

**Table 2** and **Figure 2** summarized the comparison of mediators related to angiogenesis between Y and E groups. Compared to Y group, E group had greater levels of angiostatin (p<0.05, **Figure 2A**), IL-12 (P<0.01, **Figure 2B**), and SDF1A (p<0.05, **Figure 2C**). However, the differences in the level of angiogenin, angiopoietin-1, and VEGF between groups were not statistically significant (p>0.05).

**Table 2** and **Figure 2** summarized the comparison of mediators related to tissue remodeling between Y and E groups. Y had significantly greater level of MMP-7 (p<0.05, **Figure 2D**), and less expression of MMP2 (p<0.01), MMP-3(p<0.01), OPN (p<0.001) and TIMP-1 (p<0.01) than E group. The ratios of MMP-8/TIMP-1, and MMP-9/TIMP-1 were further analyzed. Surprisingly, the ratio of MMP-9/TIMP-1 was significantly greater in Y group in comparison to E group (P<0.05, **Figure 2D**), despite there was no significant difference in MMP-8/TIMP-1 ratio between groups. MMP-1, MMP-8, MMP-9, MMP-12, MMP-13, OPG, and RANKL were also tested but did not show significant differences between groups (p>0.05, **Table 2**).

## 4 Discussion

Analysis of mediators related to host responses toward gingivitis in GCF provide valuable insights on the impacts of aging on periodontium and the disease susceptibility. Our findings showed that the younger group exhibited lower levels of inflammatory mediators compared to the elderly group, despite similar GI, PI, and PD scores between groups. Integrating data on immune regulation and tissue remodeling, younger group had less expression of MPO, IL-1β, IL-6, IL-8, and greater ratio of MMP-9/TIMP, showing lower disease susceptibility and greater tissue remodeling activities. To our knowledge, this is the first study to comprehensively compare immune and tissue responses in young and older adults with natural gingivitis.

### 4.1 Aging effects CAL and inflammatory profiles beyond plaque accumulation

Previous studies have reported age-associated changes in the periodontium following experimentally induced gingivitis (EG) in human models (4). Young adults (aged 20–25 years) exhibited fewer inflammatory cells and less pronounced gingival lesions compared to old individual (aged 65–80 years) (20). Similarly, during EG induction, young adults (aged 20–22 years) demonstrated lower plaque accumulation and bleeding scores than the old adults (aged 61–65 years) (6), which was in line with another EG study despite the difference did not reach to the statistical significance (5). In contrast to these short-term EG models, the present cross-sectional study examined the impact of age on natural gingivitis, characterized by chronic inflammation.

Our findings revealed that young adults had significantly less CAL and lower expression of inflammatory mediators than elder group although there were no significant differences found in PI, GI, and PD. It should be noted that the mean differences of CAL between group was about 0.3mm, which was statistically significant but may not be clinically significant. These results suggest that aging contributes to this aspect of periodontal destruction independent of plaque accumulation. The CAL loss in the aging population mainly results from gingival recession, instead of the PD increase from pocket formation (21). Importantly, part of the observed loss of periodontal attachment and the reduced periodontium is considered physiological rather than pathological, but can be exacerbated by the presence of periodontal inflammation (3, 22), such as gingivitis. Although the current study did not explore the microbial profiles in these gingivitis sites, the impact of aging on microbial colonization has been reported in the past decade (23, 24). In aged mice, the decrease of dendritic cell function leads to adaptive immune response in aged periodontium with periodontitis, enhancing the levels of *P. gingivalis* and reducing the bacterial diversity. This underscores the potential role of age-related alterations in immune regulation and tissue remodeling during gingivitis. To further explore these mechanisms, we analyzed biomarkers in GCF.

Prolonged exposure of the periodontium to microbial challenges may amplify age-related changes, a phenomenon known as *inflammaging* in periodontal disease. This process reduces the host ability to cope with stressors and promotes a progressively heightened intrinsic proinflammatory state during the aging process (3). In our findings, despite similar gingival inflammation (GI) scores in both the young and elder cohorts, the elder group exhibited a more complex inflammatory profile involving neutrophils, monocytes, and macrophages. Such changes may further disrupt immune homeostasis in the aged periodontium and increase susceptibility to pathogenic infection (2). These observations align with previous literature linking periodontal disease to immune senescence, the age-related decline in immune function, including altered neutrophil function and elevated inflammatory mediators such as IL-1β, IL-6, and PGE_2_ in older cohorts compared to younger patients with periodontitis (25). Interestingly, although previous EG studies have reported elevated inflammatory mediators in older population as well, such as TNF-α (6), their observation on the changes of IL-6 and MIF levels (5, 6) did not align with patterns expected from immune senescence. This discrepancy may reflect differences in inflammatory mechanisms between EG and natural gingivitis (16). The mechanisms by which aging alters immune homeostasis and influences susceptibility in the periodontium under chronic gingivitis conditions remain incompletely understood. Further investigation into immune regulation in aged gingival tissues is essential to restore innate immune function and improve oral health in older adults.

### 4.2 Aging increases susceptibility of periodontitis and decreases tissue turnover activities

Previous epidemiology studies have shown that older populations generally have greater susceptibility to periodontitis (26, 27) and our current findings further confirmed this concept with the comprehensive GCF analysis in immune and tissue responses against gingival inflammation.

Compared with the younger population, the elder group in the present study demonstrated higher levels of MPO, IL-1β, IL-6, and IL-8. Primarily released by macrophages and epithelial cells, IL-8 acts as a key chemokine that attracts and activates neutrophils (28). In healthy periodontium, the high correlation between IL-8 and MPO reflects their roles of neutrophil migration in maintaining homeostasis in periodontal tissue (29). In a diseased periodontium, the level of MPO and IL-8 also increased when disease progresses from gingivitis to periodontitis (30, 31). As the most potent proinflammatory cytokines involved in inflammatory diseases, in addition, IL-1β and IL-6 also showed a synergistical effect in human gingival fibroblasts enhancing gingival inflammation (32). Overall, the increased expression of MPO, IL-1β, IL-6, and IL-8 in old individuals may contribute to the increased susceptibility for periodontitis.

Gingiva, as a specialized mucosal immune site, represents a dynamic interface that constantly regulates immune responses while maintaining barrier integrity. Age-related declines in immunosurveillance contribute to the accumulation of senescent cells and the increase of inflammation, creating a self-reinforcing cycle that further promotes cellular senescence (33). These age-associated shifts diminish the capacity of the mucosal immune system to maintain tolerance and efficiently resolve inflammation, thereby predisposing the aged periodontium to chronic, low-grade inflammatory states. Consistent with this framework, IL-6, which correlates well with age, has been shown to drive cell senescence and to further amplify inflammatory signaling, thereby enhancing elevated local levels of pro-inflammatory cytokine and impairing the resolution of inflammation (34). Although IL-6 also possesses anti-inflammatory properties, the increased pro-inflammatory cytokine (IL-1 β and IL-8) and greater neutrophil activity (as indicated by MPO) observed in the present study may represent additional evidence of inflammaging and dysregulated innate system associated with immunosenescence. (35). Further studies are necessary to clarify the role of anti-inflammatory mediators in the pathophysiology of the aged periodontium.

Previous literature has also linked imbalances in tissue remodeling mediators, such as MMP-8 or MMP-9/TIMP ratios, to periodontal tissue destruction (36, 37). Interestingly, our results showed that younger cohorts exhibited a higher MMP-9/TIMP ratio than old individuals, whereas MMP-8/TIMP ratios did not differ significantly between groups. Despite overall low expression of tissue remodeling mediators in both groups, these findings may suggest greater tissue turnover activities in young cohorts than elder individuals, which may further enhance better healing capacity. It should also note that the elevation of pro-inflammatory cytokine have been associated with bone–remodeling activity through modulation of the RANKL–OPG axis (38). However, in the present study, the low detection of RANKL and OPG limited further interpretation, and firm conclusions cannot be drawn even though the RANKL/OPG ratio appeared higher in the aged periodontium. Future studies employing more sensitive multiplex platforms, longitudinal sampling, or targeted protein–enrichment approaches will be necessary to more accurately characterize the contributions of these mediators to bone resorption in aging tissues.

Taking both clinical and subclinical findings in the present study together, these observations provide further confirmation at synergistical effects of inflammaging and immunosenescence in aged periodontium with natural gingivitis. Along with the insignificant differences between groups in most of the clinical measurements, 0.3mm of CAL difference was not clinically meaningful, as changes of this size fell within normal measurement variability and were below thresholds used to define purely clinical framework. In the present study, the elder group exhibited subtle but consistent shifts in inflammatory mediators, such as, MPO, IL-1β, IL-6, and IL-8, reflecting subclinical host-response alterations that preceded detectable clinical deterioration. These patterns align with our broader observation suggesting lower disease susceptibility and more robust tissue remodeling activity in younger individuals, whereas older adults may experience heightened adaptive immune activation and greater subclinical inflammation despite showing comparable clinical measures. Moreover, the lower tissue turnover activities in aged periodontium may further compromise the renewal of the tissue under chronic inflammation and make it more vulnerable to the disease. Together, these findings highlight age-related differences in host biology that may influence periodontal resilience or vulnerability. Understanding these subclinical host responses could provide valuable insights into aging-related resilience factors and shape the strategies to maintain periodontal health in older adults.

### 4.3 Study limitations and future directions

Along with the inherent limitations of a cross-sectional design, this study is constrained by the lack of longitudinal data and a relatively small sample size. Consequently, the potential for disease progression in these two cohorts could not be determined, and future prospective studies are needed to evaluate the predictive value of these mediators for disease progression. Because this work represents a pilot study, it may also be underpowered; however, the present data can serve as a foundation for power calculations in subsequent research.

Although several mediators exhibited biologically meaningful differences, the levels of certain mediators (see **Supplementary Table S1**), including CCL3, MMP-2, MMP-3, RANKL, and OPG, were mostly low or below the detection range, making it difficult to calculate and compare their ratios. The results of these markers should also be interpreted with caution since the limited detectability reduces quantitative accuracy and their statistical robustness. Furthermore, the expression of these mediators does not necessarily equate to their activation at inflammatory sites, warranting caution in interpretation. It is also noted that we only assessed differences in GCF mediators between young and elderly cohorts, which primarily reflects local inflammation rather than systemic health status.

Another limitation of this study is the use of broad age categories (18–35 and 36–75 years), chosen based on the traditional 35–year threshold used in early–onset periodontitis research (39). Although practical for this pilot design, these groupings may oversimplify age–related biological variation and could contribute to methodological bias. While geriatric studies often define “older” adults as ≥65 years, our study was designed to capture early immune aging rather than geriatric frailty. Immune aging and inflammaging are well documented to begin during midlife, with measurable immunological changes occurring well before age 65. Accordingly, the elder group (≥38 years) reflects a biologically relevant stage for investigating early immune aging processes. Nevertheless, the current stratification may not optimally represent the continuum of immunosenescence, and future studies with larger cohorts should adopt more refined age groupings to better resolve age-specific immune trajectories across midlife and older adulthood.

Despite these limitations, this study provides a comprehensive analysis of GCF mediators related to inflammatory responses and tissue remodeling in the aged periodontium under chronic gingival inflammation without further disease destruction. Future research should include larger sample sizes and longitudinal, prospective designs to explore the relationships between these mediators and their causal role in disease progression. Investigations should also expand to include bacterial profiles and systemic markers, such as serum samples, to better assess the interaction between the microbiome and host responses and validate local versus systemic inflammation. Further exploration of MMP/TIMP imbalance in greater detail, along with functional assessments of aged immune cells in periodontal tissues, will provide deeper insights into age-associated changes in host responses and their impact on systemic health.

## 5 Conclusion

Within the limitations of this study, our findings demonstrate that the inflammatory profiles of young and old cohorts with natural gingivitis are distinct. In comparison to young cohort, the elder individuals exhibited higher levels of inflammatory mediators in GCF despite similar clinical indicators of inflammation. The profiles of host responses, integrating the profiles of immune regulation and tissue remodeling, suggested greater disease susceptibility and lower tissue turnover activities in aged periodontium with natural gingivitis.

Advancing knowledge on age-associated changes in immune and tissue responses to chronic bacterial challenges will be critical for developing personalized strategies aimed at restoring immune and tissue homeostasis and improving health outcomes in older adults. Future studies should prospectively monitor the interplay between immune and tissue remodeling activities in the aged periodontium to tailor precision approaches in individualized periodontal care.

## Data Availability

The original contributions presented in the study are included in the article/**Supplementary Material**. Further inquiries can be directed to the corresponding author.

## Supplementary material

Supplementary Table 1 Detection Summary of GCF Analytes Across Study Cohorts. CRP, C-reactive protein; GM-CSF, Granulocyte-macrophage colony-stimulating factor; ICAM-1, intercellular adhesion molecule-1; IL, Interleukin; MMP, matrix metalloproteinase; MPO, myeloperoxidase; OPN, osteopontin; OPG, osteoprotegerin; RANKL, Receptor activator of nuclear factor-κB ligand; TIMP, metallopeptidase inhibitor; VEGF, Vascular endothelial growth factor.
