# Supplementary material for: Effects of aging on host responses in gingival crevicular fluid in natural gingivitis
Source: Front Immunol. 2026 Feb 26;17:1761707. doi: 10.3389/fimmu.2026.1761707 (PMC12979528; doi:10.3389/fimmu.2026.1761707)
Supplement: Supplementary Table 1 — Detection Summary of GCF Analytes Across Study Cohorts. CRP, C-reactive protein; GM-CSF, Granulocyte-macrophage colony-stimulating factor; ICAM-1, intercellular adhesion molecule-1; IL, Interleukin; MMP, matrix metalloproteinase; MPO, myeloperoxidase; OPN, osteopontin; OPG, osteoprotegerin; RANKL, Receptor activator of nuclear factor-κB ligand; TIMP, metallopeptidase inhibitor; VEGF, Vascular endothelial growth factor. [file Table1.docx]

**Table S1. Detection Summary of GCF Analytes Across Study Cohorts.**

|  | **Young (Y)**  **(n=22)** | **Elder (E)**  **(n=18)** | **Limit of detection** |
| --- | --- | --- | --- |
| LOX_1 | 21 | 18 | 0.02-6300 |
| IL-1B | 17 | 18 | 0.03-7000 |
| IL_6 | 12 | 15 | 0.16-42000 |
| IL_8 | 18 | 18 | 0.04-9250 |
| IL_17A | 19 | 18 | 0.04-11100 |
| MIF | 22 | 18 | 0-1100 |
| CCL3 | 3 | 8 | 0.04-9600 |
| MPO | 18 | 18 | 1.31-344100 |
| ICAM_1 | 17 | 18 | 1.91-501200 |
| CCL5/RANTES | 20 | 18 | 0.01-3500 |
| Fractalkine/CX3CL1 | 11 | 16 | 0.02-5750 |
| GM_CSF | 10 | 11 | 0.19-49900 |
| CCL2 | 14 | 15 | 0.08-20000 |
| C3a | 18 | 18 | 0.54-142800 |
| CRP | 21 | 18 | 0.11-28800 |
| ENA-78/CXCL5 | 9 | 14 | 0.11-29100 |
| CXCL6 | 9 | 11 | 0.02-6200 |
| Angiostatin | 22 | 18 | 0.19-5150 |
| IL_12p70 | 18 | 18 | 0.12-32300 |
| SDF_1_alpha | 11 | 14 | 0.24-63400 |
| Angiogenin | 11 | 18 | 0.01-600 |
| Angiopoietin-1 | 8 | 11 | 7.08-145600 |
| VEGF_A | 11 | 16 | 0.08-20000 |
| MMP_1 | 19 | 18 | 0.11-28800 |
| MMP_2 | 8 | 13 | 0.12-32700 |
| MMP_3 | 15 | 18 | 0.03-7100 |
| MMP_7 | 21 | 18 | 0.08-21200 |
| MMP_8 | 22 | 18 | 0.39-101700 |
| MMP_9 | 22 | 18 | 0.02-4400 |
| MMP_12 | 8 | 11 | 0.1-25100 |
| MMP_13 | 14 | 18 | 0.12-32500 |
| TIMP_1 | 19 | 18 | 0.44-116300 |
| OPN | 8 | 18 | 0.28-73300 |
| OPG | 8 | 11 | 0.01-2800 |
| RANKL | 10 | 10 | 0.12-30500 |
| BMP-2 | 11 | 15 | 1.16-303000 |
| Insulin | 19 | 18 | 728.69-860207.89 |
| c-peptide | 18 | 18 | 1.13-295700 |

CRP= C-reactive protein, GM-CSF= Granulocyte-macrophage colony-stimulating factor, ICAM-1= intercellular adhesion molecule-1, IL= Interleukin, MMP= matrix metalloproteinase, MPO= myeloperoxidase, OPN= osteopontin, OPG= osteoprotegerin, RANKL= Receptor activator of nuclear factor-κB ligand, TIMP= metallopeptidase inhibitor, and VEGF= Vascular endothelial growth factor
